# Finger-Stick Whole Blood HIV-1/-2 Home-Use Tests Are More Sensitive than Oral Fluid-Based In-Home HIV Tests

**DOI:** 10.1371/journal.pone.0101148

**Published:** 2014-06-27

**Authors:** Marie Jaspard, Gwenaël Le Moal, Mariam Saberan-Roncato, David Plainchamp, Aurélie Langlois, Pascale Camps, Aurélie Guigon, Laurent Hocqueloux, Thierry Prazuck

**Affiliations:** 1 CHR d'Orléans – La Source, Infectious and Tropical Diseases Department, Orléans, France; 2 CHU de Poitiers, Infectious and Tropical Diseases Department, Poitiers, France; 3 CH de La Rochelle, Infectious Diseases Department, La Rochelle, France; 4 CHR d'Orléans – La Source, Laboratory of Microbiology, Orléans, France; New York University, United States of America

## Abstract

**Background:**

Several countries have recently recommended the expansion of anti-human immunodeficiency virus (HIV) antibody testing, including self-testing with rapid tests using oral fluid (OF). Several tests have been proposed for at-home use, but their diagnostic accuracy has not been fully evaluated.

**Objective:**

To evaluate the performance of 5 rapid diagnostic tests for the detection of anti-HIV-1/2 antibodies, with 4 testing OF and 1 testing whole blood.

**Methods:**

Prospective multi-center study in France. HIV-infected adults and HIV-uninfected controls were systematically screened with 5 at-home HIV tests using either OF or finger-stick blood (FSB) specimens. Four OF tests (OraQuick Advance Rapid HIV-1/2, Chembio DPP HIV 1/2 Assay, test A, and test B) and one FSB test (Chembio Sure Check HIV1/2 Assay) were performed by trained health workers and compared with laboratory tests.

**Results:**

In total, 179 HIV-infected patients (M/F sex ratio: 1.3) and 60 controls were included. Among the HIV-infected patients, 67.6% had an undetectable HIV viral load in their plasma due to antiretroviral therapy. Overall, the sensitivities of the OF tests were 87.2%, 88.3%, 58.9%, and 28% (for OraQuick, DPP, test A, and test B, respectively) compared with 100% for the FSB test Sure Check (p<0.0001 for all comparisons). The OraQuick and DPP OF tests' sensitivities were significantly lower than that of the FSB-based Sure Check (p<0.05). The sensitivities of the OF tests increased among the patients with a detectable HIV viral load (>50 copies/mL), reaching 94.8%, 96.5%, 90%, and 53.1% (for OraQuick, DPP, test A, and test B, respectively). The specificities of the four OF tests were 98.3%, 100%, 100%, and 87.5%, respectively, compared with 100% for the FSB test.

**Conclusion:**

An evaluation of candidates for HIV self-testing revealed unexpected differences in performance of the rapid tests: the FSB test showed a far greater reliability than OF tests.

## Introduction

A lack of knowledge regarding human immunodeficiency virus (HIV) status is a public health issue, especially in resource-limited settings, such as developing countries. Many people living with AIDS, including 60% of those living in resource-limited countries, are unaware of their HIV status [1]. In France, approximately 30,000 people do not know they are infected with HIV [2]. This lack of knowledge regarding HIV status can increase the risk of transmission within the general population and may compromise the success of new prevention strategies, such as oral pre-exposure and post-exposure prophylaxis and microbicide gel. In addition, approximately 40% of new diagnoses are made during a late stage of infection when patients are already severely immunosuppressed [3], [4], leading to increased mortality [5].

In France, rapid HIV tests using blood samples are currently implemented at health care centers for professional use and have been available to trained volunteers working in community AIDS associations since 2010. In England, rapid tests are well accepted by the population [6], as these tests are easy to use and can be performed by trained staff, with results obtained within a few minutes [7]. However, these tests are currently only available at certain medical facilities, and the French AIDS National Council (CNS) recently recommended that their use should be expanded [8]. Some men having sex with men (MSM) admit that they administer self-tests that are sold illegally on websites, indicating that autonomous self-testing may reduce barriers to testing in this vulnerable population [9]. The USA has recently authorized the marketing and commercialization of the over-the-counter OraQuick OF in-home test, which can be used without any prior training or assistance from health professionals. In France, the National Ethic Committee and National AIDS Council (CNS) recently approved the implementation of self-testing for HIV diagnosis, provided that the test accuracy is deemed acceptable through public health policy [8],[10].

Because of the simplicity and safety of OF collection compared to FSB collection, OF-based tests are well accepted as in-home tests. It has been two decades since the salivary rate of HIV antibodies was first evaluated. However, the accuracy of these tests varies among published studies; for example, the OraQuick OF test showed a sensitivity that ranged from 86% to 100% [7], [11]–[13]. Indeed, immunoglobulin G (IgG) is present in OF, especially crevicular fluid, but its concentration is nearly 800 times lower than that found in the serum [14], [15]. The post-marketing surveillance of OraQuick whole blood and oral fluid rapid testing indicated that the specificity of this test was lower than the range indicated in the package insert [16].

The aim of this study was to compare the diagnostic accuracy (sensitivity and specificity) of 4 OF tests and one FSB test, all of which are specifically designed for in-home testing.

## Methods

### Study design and patient characteristics

The study was conducted from January to July 2013 in 3 French health centers: La Rochelle, Poitiers and Orleans. The patient group consisted of 179 known HIV-infected subjects either being seen for their medical follow-up or being hospitalized in the Infectious Disease Department (IDD). The control group included HIV non-infected individuals who received consultations at a free anonymous screening center or were hospitalized in the IDD for another medical reason. All 5 tests were administered to all patients, and written informed consent was obtained. This study was approved by the Orleans Hospital ethics committee.

The characteristics of each patient were collected and reported on a medical form (including age, sex, geographic origin, CDC stage, HIV and subtype, antiretroviral therapy and CD4 cell count). Viral load was determined using a Cobas Taqman assay (Roche Industry, France). A fourth-generation EIA utilizing the p24 antigen, the Architect Combo HIV-1/-2 Assay Abbott, was considered the gold standard and performed on a blood sample from each patient.

### HIV rapid tests designed for self-testing

A total of 5 rapid HIV tests, 4 using OF and 1 using FSB specimens, were administered to both groups of patients by trained medical staff in the following order: OraQuick Advance Rapid HIV-1/-2 Antibody Test (OraSure Technologies, Bethlehem, PA, USA); DPP HIV-1/-2 Assay (Chembio Diagnostic Systems, Medford, NY, USA); test A (HIV-1/-2 Oral Fluid, USA); test B (HIV-1/-2 Whole Saliva Test, USA); and the FSB Sure Check HIV-1/-2 Assay (Chembio Diagnostic Systems, Medford, NY, USA) using 2.5 µl FSB. All tests were performed according to the manufacturer instructions, and the characteristics of each test are summarized in Table 1.

**Table 1:** Technical characteristics of the HIV rapid screening tests.

Test	Manufacturer	Type of sample	Principle and HIV antigens	Time to result	No. of steps to perform the test	Reported accuracy
OraQuick Advance Rapid HIV-1/-2 Antibody Test	OraSure Technologies, USA	Crevicular fluid (pad) 5 µl	Immunochromatography HIV-1 (gp41) and HIV-2 (gp36)	20 to 40 min	4	Se 100% and Sp 99.8%
DPP HIV-1/-2 Assay	Chembio Diagnostic Systems, USA	Crevicular fluid (pad) 5 µl	Immunochromatography HIV-1 (gp41 and gp120) and HIV-2 (gp36)	15 to 30 min	5	Se 100% and Sp 99.9%
Test A	Nondisclosure agreement, USA	Crevicular Fluid	Immunochromatography HIV-1 (gp41) and HIV-2 (gp36)	20 to 45 min	5	Se and Sp 100%
Test B	Nondisclosure agreement, USA	Whole saliva	Immunochromatography HIV-1 (gp41) and HIV-2 (gp36)		14	Se 99.7% and Sp 99.9%
Sure Check HIV-1/-2 Assay	Chembio Diagnostic Systems, USA	Blood 2.5 µl	Immunochromatography HIV-1 (gp41 and gp120) and HIV-2 (gp36)	15 to 20 min	4	Se 99.7% and Sp 99.9%

Abbreviations: Se  =  sensitivity; Sp  =  specificity.

The selection of these five tests was based on the fact that they have been designed and manufactured for both home testing and professional use. However, the FSB OraQuick was not included in this study because, similar to the OF OraQuick (the only test approved for HIV self-testing in the USA), it does not permit capillary blood collection for in-home test. Additionally, the INSTI test (Biolytical Inc., Canada) was not included in this study because it is only recommended for professional use and requires 50 µL of capillary blood, making it unsuitable for self-testing or administration by untrained personnel.

The number of steps required for HIV self-testing varied from 4 to 14, including sampling and reading. All tests were read twice, first by the investigator who administered the test and then by a second investigator who was blinded to the serological status of the patient. The results were recorded as positive (including weakly positive), negative, invalid (no control band), or impossible to perform (in the case of test B) due to difficulty with sample collection.

The delay to obtain a positive result following fluid collection was measured using a chronometer and recorded for each patient. The result was always read at the time recommended by the manufacturer.

Health workers involved in the study received a one-day training session for each test prior to the beginning of the study. However, all nurses were already highly experienced with these tests, as HIV rapid tests and INSTI HIV rapid tests had been used routinely for several years at our centers.

The level of practicability was assessed by the health workers involved in the study based on five criteria: the clarity of the manufacturer's instructions, estimated risk to fail, complexity of the procedure, concordance between readers and estimated practicability for the untrained user. Each item was divided into three categories (fair, acceptable and poor), and an aggregate score ranging from 0 to 5 was determined for each test by each operator.

### Statistical analysis

Sensitivity was calculated by dividing the number of positive and weakly positive tests by the number of valid tests for each patient group. The number of negative tests in the control group was divided by the number of valid tests to determinate the specificity of each test. Furthermore, test sensitivity estimates were stratified by viral load and CD4 T cells counts. The 95% confidence interval (CI) was calculated for each group of patients, and the P value was calculated using the Chi^2^ test.

## Results

A total of 179 HIV-infected patients and 60 HIV non-infected patients were included. Among the HIV-infected patients, 65% were male and 70% were Caucasian. In the HIV non-infected group, 33% were male and 98% were Caucasian. Only 2 patients were HIV-2-positive, and 44% of the HIV-1-positive patients were infected with the subtype B virus. Among the HIV-infected patients, 49% (88/179) had been infected with HIV for over 10 years, and 60% (107/179) were at stage A of the CDC score. Furthermore, 73% (131/179) had received HAART for at least 2 years. The median CD4 value was 533/mm^3^, and 53% (95/179) of patients presented CD4 counts above 500/mm^3^. Finally, 67% (121/179) of the infected patients had an undetectable viral load (<50 cp/mL).

Tests were performed on both the HIV-infected and non-infected patients. A total of 238 patients were tested with OraQuick, the DPP OF test and Sure Check; 205 patients were tested with test A; and 218 patients were tested with test B.

The sensitivity and specificity of each test are summarized in Table 2.

**Table 2:** Accuracy of 4 OF tests (OraQuick, DPP, Test A, Test B) and 1 FSB test (Sure Check) as compared with Architect Combo HIV-1/-2 Assay Abbott (reference test).

	Sensitivity	95% CI	Specificity	95% CI
**OraQuick**	87.2%	[81.5–91.3]	98.3%	[91.1–99.7]
**DPP**	88.3%	[82.7–92.2]	100%	[94–100]
**Test A**	58.9%	[51.1–66.2]	100%	[94–100]
**Test B**	28%	[21.3–35.8]	87.5%	[76.4–93.8]
**Sure Check**	100%	[97.9–100]	100%	[94–100]

Footnote: false negative rates are 12.8%, 11.7%, 41.1%, 72% and 0% for OraQuick, DPP, test A, test B and Sure Check tests, respectively.

In the study population, 23 false-negative results were observed using OraQuick and 21 using the DPP OF tests. For the OraQuick test, these false-negative results corresponded to 11/23 patients who had received an HIV diagnosis >10 years ago, 23/23 who were receiving HAART, 18/23 who had been receiving HAART for >2 years, 20/23 with an HIV viral load <50 copies/mL and 14/23 with CD4 T cell counts >500/mm^3^. For the DPP OF test, these false-negative results corresponded to 12/21 patients who had received an HIV diagnosis >10 years ago, 21/21 who were receiving HAART, 19/21 who had been receiving HAART for >2 years, 19/21 with an HIV viral load <50 copies/mL and 13/21 with a CD4 T cell count >500/mm^3^. All of the false-negative results using the DPP OF test also showed false negatives using the OraQuick OF test.

The sensitivity and 95% CI for each test according to the HIV viral load detectability (< or >50 copies/mL) and CD4 T cell count (> and <500/mm^3^) are reported in Table 3. Overall, there are trends suggesting that at lower viral loads and higher CD4 counts, the oral fluid based tests have lower sensitivities. However, the Sure Check HIV test sensitivity was 100% in each of these categories. Furthermore, although the recommended reading time for this FSB test is 15–20 minutes, all positive results were obtained within 2 minutes.

**Table 3:** HIV self-test sensitivity (95% CI) according to HIV-1 viral load and CD4 T cell count.

	VL <50 cp/mL n = 121	95% CI	VL >50 cp/mL n = 58	95% CI	CD4 >500/mm^3^ n = 95	95% CI	CD4 <500/mm^3^ n = 84	95% CI
**OraQuick**	83.5%	[75.8–89]	94.8%	[85.8–98.2]	85.2%	[76.7–91]	89.3%	[80.9–94.2]
**DPP**	84.3%	[76.7–89.7]	96.5%	[88.2–99]	86.3%	[78–91.8]	90.5%	[82.3–95.1]
**Test A**	48.3%	[39.5–57.2]	90.0%	[76.9–96]	48.8%	[38.5–59.2]	70.8%	[59.5–80.1]
**Test B**	20.7%	[14.2–29.2]	53.1%	[36.4–69.1]	17.9%	[11–27.9]	40%	[29–52.1]
**Sure Check**	100.0%	[96.9–100]	100.0%	[93.8–100]	100%	[96.1–100]	100%	[95.6–100]

Abbreviations: VL  =  plasma viral load.

Practicability estimated by the trained nurses and measured by the aggregate score was 15/15 for the OraQuick, DPP OF and Sure Check tests, 13/15 for test A and 5/15 for test B.

## Discussion

Usually, sensitivity performance of commercialized HIV rapid tests, as indicated in their package inserts, are excellent and very close to 100%. Our study was carried out by three university medical schools independently of any of the four manufacturers, and detected sensitivity values below 96% for the OF-based tests compared to up to 100% for the FSB test. The differences between the sensitivity ranges indicated in the package inserts and the results obtained in this study highlight the need for quality assurance procedures, as previously mentioned [16].

The FSB Sure Check test demonstrated a sensitivity and specificity of 100% (95% CI: 95.6–100%). FSB tests have been evaluated in previous studies and have been shown to be highly sensitive [7], [13]. The most sensitive HIV test currently in use in France, the INSTI, is not designed for in-home testing because it requires collecting 50 µl of blood, which is not feasible for an untrained individual. In our study, the OraQuick and DPP OF tests exhibited sensitivity values of 87.2% and 88.3%, respectively.

OraQuick is the only OF test available on the US market, and several accuracy evaluation studies have reported variable results. For example, a study in Zimbabwe reported 100% sensitivity in patients presenting for screening who had not previously received HAART [11]. Another study performed in HIV-naïve patients in Zambia revealed a 98.7% sensitivity (95% CI: 97.5–99.4%) for the OraQuick Advance Rapid HIV-1/-2 antibody test [17]. Two additional studies performed on patients receiving HAART demonstrated high sensitivity for the OraQuick test, including 97.8% in Spain [12] and 99.3% in the US [13]. However, a recent French study reported a lower sensitivity for the OraQuick test (86.7%) [7].

In agreement with previous reports by Pavie et al. in a population of treated HIV patients with low viral loads and high CD4 cell counts [7], our findings show a lower sensitivity for OF tests compared to an FSB test. It is important to note that even with a detectable HIV viral load, the sensitivity of OF tests is reduced in HIV patients receiving HAART. In particular, a US study suggested that HAART may decrease gp41 production, which is the test antigen used to detect HIV antibodies in the OraQuick assay [18]. However, the Sure Check FSB and DDP Chembio OF tests include gp120 in addition to gp41, which could increase their accuracy under these conditions. This difference may also explain the lower sensitivity of certain OF tests in treated patients. The present study confirmed the difficulty associated with performing HIV test evaluations on a significant number of untreated patients in countries where most infected patients receive HAART.

Tests that yield a significant rate of false-negative results in treated HIV patients may lead to dangerous situations. For example, it is possible that a patient who is aware of his HIV-positive status may choose to buy a rapid test and perform it at home. A false “negative” Result could trigger a disbelief in the original diagnosis provided by the physician. Nevertheless, in the Western World, individuals with HIV, receiving HAART and viral load monitoring, are not very likely to buy and use a rapid HIV OF antibody tests.

Although the practicability of the evaluated tests was well acknowledged by the study nurses, further work is needed to evaluate self-tests performed by untrained subjects. Because proper test interpretation and understanding of the results might be challenging for certain individuals who are experiencing a high level of stress, the availability of trained staff to help the patient as needed could be useful.

The principle and methodology of HIV self-testing should be discussed for both ethical and financial reasons. It is not assumed that pre-test or post-test counseling will be provided, although a study performed in Asia found that 73% of patients were willing to receive a professional consultation [19]. It has also been reported that unskilled individuals are equally able to perform these HIV tests on themselves compared to trained medical staff [19]–[21], with a preference for OF tests [21]. Self-test acceptability was shown to be good [21], [22], although high prices pose a problem even in developed countries [20], with only 28% of patients willing to pay 15 US dollars for a test [19]. Moreover, the vast majority of HIV-infected subjects live in low-resource countries where in-home tests could be an option only if they were affordable for the population. However, the confidentiality of self-test results is a major asset of this approach, which would give more independence to people who are afraid to share their diagnosis while AIDS remains a stigmatizing disease.

Although OF-based self-tests offer an attractive approach for additional HIV screening, new-generation FSB tests offer both a reliability near 100% and excellent specificity. FSB tests are now well known, widely accepted and highly efficient. In particular, the Sure Check test was shown to be as easy to use as the OF OraQuick and Chembio DPP tests and only requires a small sample of blood (2.5 µl). The development of a global strategy for home-test availability is necessary to ensure that these self-tests will be properly used and that newly HIV-diagnosed patients will enter the health care system for HIV diagnosis confirmation and treatment, facilitating a reduction in the spread of HIV infection. Therefore, a study to evaluate the practicability and reading capability of the Sure Check test for individuals receiving HIV testing is now ongoing.
